# Emergence of a Recombinant Bovine Enterovirus in China: Insights from Phylogenetic and Temporal Analysis

**DOI:** 10.3390/ani15101457

**Published:** 2025-05-18

**Authors:** Guidan Feng, Taisheng Kang, Pan Tang, Caihua Xie, Ruoqian Yan, Weidong Qian

**Affiliations:** 1 Shanghai Animal Disease Prevention and Control Center, Shanghai 201103, China; f188603@126.com (G.F.); kangtaisheng11@126.com (T.K.); 2 Institute of Animal Husbandry and Veterinary Medicine, Shanghai Academy of Agricultural Sciences, Shanghai 201106, China; tangpan8811@163.com; 3 Henan Centre for Animal Disease Prevention and Control, Zhengzhou 450008, China; xiecaihua8301@126.com; 4 School of Biological and Pharmaceutical Sciences, Shaanxi University of Science and Technology, Xi’an 710021, China

**Keywords:** bovine enterovirus, diarrhea, molecular characterization, divergence dating, recombination breakpoints

## Abstract

Bovine enteroviruses (BEVs) are emerging pathogens with poorly understood evolutionary dynamics and zoonotic potential. Here, we report the discovery of a novel recombinant BEV strain, HeN-2022, isolated from cattle in China. Genomic analysis revealed that HeN-2022 is a primary hybrid of BEV-E1 (VG527, Ireland) and BEV-E4 (GX1901, China), with recombination breakpoints in the VP1 gene and 5′ UTR. Divergence dating traced its origin to 1991, predating closely related strains. Experimental infection in sheep demonstrated asymptomatic viral shedding (peak at 5 dpi) and robust neutralizing antibody responses, highlighting the potential cross-species adaptability. These findings underscore recombination as a potential key driver of BEV evolution and emphasize the need for global surveillance to address emerging livestock pathogens.

## Simple Summary

Bovine enteroviruses (BEVs) are viruses that can cause health problems in cows, such as diarrhea and respiratory illness. These viruses can also spread to other animals and potentially to humans. In this study, a new type of BEV called HeN-2022 in China was reported. This virus is a mix of two other BEV types and has been around since 1991. The study aimed to understand how this isolate formed and how it might affect animals. We tested the isolate on sheep and found that it could spread without causing obvious symptoms, but it did trigger an immune response. This means the virus could be more widespread than we think. The findings highlight the importance of monitoring these viruses to prevent outbreaks and protect both animal and human health. Understanding how these viruses evolve and spread helps us develop better strategies to control them and reduce their impact on livestock industries.

## 1. Introduction

Bovine enteroviruses (BEVs), members of the *Picornaviridae* family, are classified into two species: Enterovirus-E (EV-E, subtypes E1–E4) and Enterovirus-F (EV-F, subtypes F1–F6). These non-enveloped, icosahedral viruses (25–30 nm in diameter) possess a single-stranded [1], positive-sense RNA genome (7.5 kb) that encodes a polyprotein. Proteolytic processing of this polyprotein yields four structural capsid proteins (VP1–VP4) and seven nonstructural proteins involved in replication and pathogenesis [2,3]. Notably, the VP1 protein, which forms part of the capsid’s “canyon” domain, is critical for host cell receptor binding and viral tropism, with amino acid variations directly influencing pathogenicity [4]. Despite advances in understanding enterovirus biology, the cellular receptor for BEV remains unidentified.

First isolated in the 1950s, BEVs are endemic in major cattle-rearing regions, including the United States, Canada, Europe, and China [2,5,6,7]. Infections are often subclinical but may manifest as diarrhea or respiratory illness, posing economic risks to livestock industries. BEVs exhibit broad host adaptability, with documented infections in sheep, moose, and even marine mammals [8]. Notably, human fecal and blood samples have occasionally tested positive for BEV, raising concerns about zoonotic transmission [9]. In China, BEV was first reported in 1994, and subsequent surveillance has revealed a prevalence of 4.5–45.5% across provinces [10,11], with significant genetic diversity among circulating strains [12].

Recombination, a hallmark of enterovirus evolution, drives the emergence of novel variants with altered host ranges and virulence. The advent of next-generation sequencing has uncovered extensive BEV genetic heterogeneity, underscoring its capacity for cross-species adaptation and regional spread. However, although the clinical manifestations of BEV have been widely reported, their pathogenicity pales in comparison to that of other bovine enteric pathogens, such as bovine viral diarrhea virus. Regrettably, comprehensive investigations into the intricate molecular mechanisms of BEV, including receptor binding and immune evasion, remains limited [6,13,14].

In this study, we isolated a novel BEV strain, HeN-2022, from diarrheic cattle in Henan Province, China. Through whole-genome sequencing, phylogenetic reconstruction, and divergence time estimation, we elucidated its recombinant origins and evolutionary trajectory. Furthermore, experimental infections in sheep revealed asymptomatic viral shedding and seroconversion, providing insights into cross-species transmission potential. Our findings advance the understanding of BEV evolution and underscore the need for proactive surveillance to mitigate emerging threats in global livestock populations.

## 2. Materials and Methods

### 2.1. Sample Collection and Nucleic Acid Extraction

From October 2021 to July 2022, a total of 156 fecal samples were collected from cattle exhibiting acute clinical symptoms, such as diarrhea, high fever, and lethargy, as well as from cattle without diarrhea, across five cattle farms in Henan Province, China. Fresh fecal specimens were diluted 1:5 (*w*/*v*) in sterile phosphate-buffered saline (PBS, pH 7.4) and stored at −80 °C until processing. Total RNA was extracted using the QIAamp Viral RNA Mini Kit (Qiagen, Hilden, Germany), following the manufacturer’s protocol. Complementary DNA was prepared from 1 μg of RNA using the 5× PrimeScript RT Master Mix (Takara, Dalian, China).

### 2.2. Reverse Transcription-Polymerase Chain Reaction (RT-PCR) and Sequencing

Screening for BEV was performed via RT-PCR using previously validated primers targeting conserved regions of the viral genome [15]. Positive samples were subjected to full-genome amplification using five overlapping primer pairs (Table S1), synthesized by General Biology Co. (Dalian, China). RT-PCR products were purified using a gel extraction kit (Qiagen), cloned into the pGEM-T vector (Promega, Madison, WI, USA), and transformed into competent *E. coli* DH5α cells. Plasmid DNA was sequenced on an ABI Prism 3730xl DNA sequencer (Applied Biosystems, Waltham, MA, USA). Consensus sequences were assembled using SeqMan Pro 12.2 (DNASTAR, Madison, WI, USA) and compared to GenBank entries via BLAST.

### 2.3. Virus Isolation and Propagation

Madin-Darby bovine kidney (MDBK) cells were cultured in Dulbecco’s Modified Eagle Medium (DMEM; Gibco, Waltham, MA, USA) supplemented with 10% fetal bovine serum (FBS), 100 U/mL penicillin, and 100 μg/mL streptomycin at 37 °C under 5% CO_2_. Fecal supernatant filtrate was treated with 25 μg/mL trypsin (37 °C, 1.5 h) to activate viral particles. Cultured monolayers of MDBK cells were cultured with the treated supernatant for 1 h, washed thrice with PBS, and kept in serum-free DMEM medium with trypsin (4 μg/mL) [16]. Cytopathic effects (CPE), characterized by cell rounding and detachment, were evaluated for 72 h. Virus-containing supernatants from passages showing >80% CPE were harvested, aliquoted, and stored at −80 °C.

### 2.4. Transmission Electron Microscopy (TEM)

Viral particles were cellected from virus-infected cell filtrates by polyethylene glycol (PEG 8000, Thermo Fisher Scientifi, Beijing, China) precipitation and ultracentrifugation (11,000× *g*, 30 min). Pellets were resuspended in deionized water and incubated overnight at 4 °C with anti-BEV polyclonal serum (1:1 ratio). The complexes were pelleted (16,000× *g*, 1 h), resuspended in 50 μL deionized water, and treated with 2% phosphotungstic acid. The samples were examined under a Hitachi HT7800 TEM (Hitachi, Tokyo, Japan) at 80 kV.

### 2.5. Viral Growth Kinetics

The 50% tissue culture infectious dose (TCID_50_/mL) of HeN-2022 was determined in MDBK cells using 10-fold sequential dilutions. Cells were assessed for CPE at 48 h post-infection (hpi), and titers were calculated. For growth-curve analysis, MDBK cells were infected at a multiplicity of infection of 0.05. Supernatants were gathered at 12, 24, 36, 48, and 72 hpi, and viral titers were plotted as the mean ± SD from three independent experiments.

### 2.6. Genomic and Phylogenetic Analysis

The complete genome of HeN-2022 was annotated using DNASTAR 7.0. Nucleotide sequences were aligned with global BEV strains (GenBank) via ClustalW in MegAlign. Phylogenetic trees were constructed using the neighbor-joining method with 1000 bootstrap replicates in the MEGA 11.0 software. Divergence times were estimated via the RelTime-Dated Tips method, incorporating BEV strains isolated between 1956 and 2022 (Table S2).

### 2.7. Recombination Analysis

To contextualize these findings within the broader landscape of global BEV diversity, we integrated a curated dataset comprising publicly available BEV genomes from the NCBI GenBank and Virus Pathogen Resource databases. These sequences, which span six decades (1956–2022) and represent 23 countries, enabled the comparative analysis of recombination hotspots and lineage-specific evolutionary trends. We characterized recombination in the HeN-2022 genome using RDP4 (v4.101) with seven built-in algorithms (RDP, GENECONV, BootScan, MaxChi, Chimaera, SiScan, PhylPro), applying a 200 bp sliding window (20 bp step) and Bonferroni-corrected “*p*” < 0.05 thresholds, with potential events requiring consensus from ≥4 algorithms. Breakpoints were validated through RDP4’s integrated BootScan viewer (1000 replicates) and similarity plots comparing HeN-2022 to parental references [17]. 

### 2.8. Animal Challenge Experiments

Twenty-four healthy sheep (12 adults: 24 months; 12 lambs: 12 months) were obtained from the Henan Centre for Animal Disease Control. The animals were kept in temperature-regulated facilities with free access to food and water. The animals were randomly divided into three groups:

- Oral inoculation (*n* = 8): 10^5^ TCID_50_/mL of HeN-2022.
- Intraperitoneal injection (*n* = 8): 10^5^ TCID_50_/mL of HeN-2022.
- Control (*n* = 8): PBS.
- Inoculation dose for each animal: 5 mL.

Fecal and blood samples were collected daily for 7 days post-infection (dpi). Viral RNA in anal swabs was quantified via RT-PCR, and antibodies in the serum were measured by indirect immunofluorescence assay (IFA). The animal challenge experiment was approved by the ethics committee of the Henan Centre for Animal Disease Control.

### 2.9. IFA

MDBK cells infected with HeN-2022 were fixed with ice-cold methanol:acetone (1:1) for 30 min at 4 °C. After pre-treatment with 5% bovine serum albumin (BSA), the cells were incubated at 37 °C for 1 h. The cells were rinsed thrice with PBS and exposed to Fluorescein isothiocyanate-conjugated mouse anti-goat IgG (1:200; Sigma, St. Louis, MO, USA) for 1 h in darkness. Fluorescence signals were presented visually using an Olympus IX73 microscopy (Olympus Corporation, Tokyo, Japan).

## 3. Results

### 3.1. Epidemiological Prevalence of BEV in Henan Province

A total of 156 fecal samples were collected from diarrheic and non-diarrheic cattle across five farms in Henan Province. RT-PCR screening revealed a BEV positivity rate of 19.2% (30/156), with no significant difference between diarrheic (19.4%, 21/108) and non-diarrheic (18.75%, 9/48) groups (*p* > 0.05, chi-square test). Four of the five surveyed farms tested positive, potentially indicating widespread BEV circulation in the region.

### 3.2. Isolation and Characterization of HeN-2022

**Figure 1:**
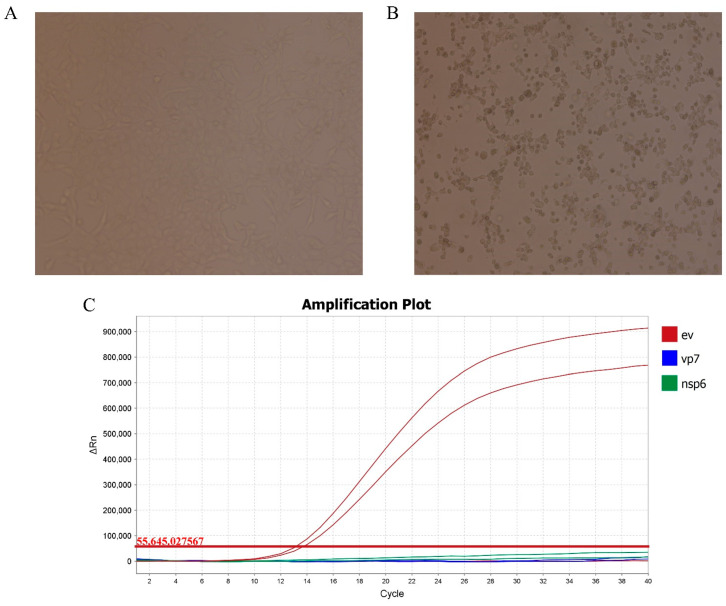
(**A**) MDBK cells untreated with HeN-2022; (**B**) CPE caused by HeN-2022 in MDBK cells was characterized; (**C**) HeN-2022 were identified by RT-PCR.

**Figure 2:**
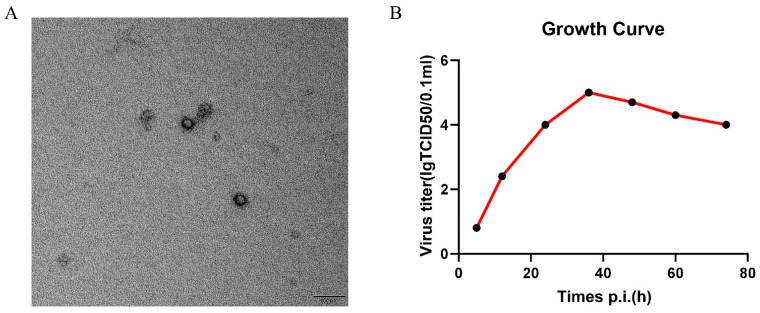
(**A**) TEM examination of HeN-2022; (**B**) Growth curve of HeN-2022 in MDBK cells.

Compared with the uninfected cells that feature a neat and regular structure (Figure 1A, virus isolation in MDBK cells induced CPE within 12 h post-inoculation (hpi), characterized by cell rounding, aggregation, and detachment (Figure 1B). Serial passaging (five generations) confirmed stable viral replication, with RT-PCR yielding a low cycle threshold value of 12, indicative of a high viral load (Figure 1C). The TEM of purified supernatants revealed non-enveloped, icosahedral particles (27–30 nm) consistent with *Picornaviridae* morphology (Figure 2A). Growth kinetics analysis demonstrated a rapid replication cycle, with titers peaking at 10^5^ TCID_50_/mL by 36 hpi and declining to 10^4^ TCID_50_/mL at 72 hpi (Figure 2B). The complete genome of HeN-2022 (7416 nt, GenBank: OR058627) comprises a 5′ UTR (812 nt), a single polyprotein ORF (6528 nt; positions 813–7341), and a 3′ UTR (76 nt). The polyprotein (2176 aa, ~242.7 kDa) shares 66.9–80.5% nucleotide identity and 50.1–79.2% amino acid identity with global BEV strains, showing the closest homology to the BEV-E4 strain GX1901 (China, 80.5%).

In addition, the virulence stability characteristics of the HeN-2022 strain of BEV were examined during cell passaging. Following primary virus isolation, an initial adaptation phase was observed during the first three serial passages in MDBK cells. This phase was characterized by genomic instability and phenotypic variability, which is consistent with the common observation that viruses often undergo an adaptation period when introduced to a new cell culture environment. In contrast, from passage 4 to 45, the HeN-2022 strain entered a plateau phase of virulence stabilization. During this period, no statistically significant attenuation was detected in pathogenicity experiments (*p* > 0.05). Our experimental data further demonstrate that the virulence attenuation threshold occurs beyond passage 50, as evidenced by a 62.3 ± 5.1% reduction in the in vitro CPE at passage 50 compared to passage 40.

### 3.3. Phylogenetic Classification and Recombination Events

**Figure 3:**
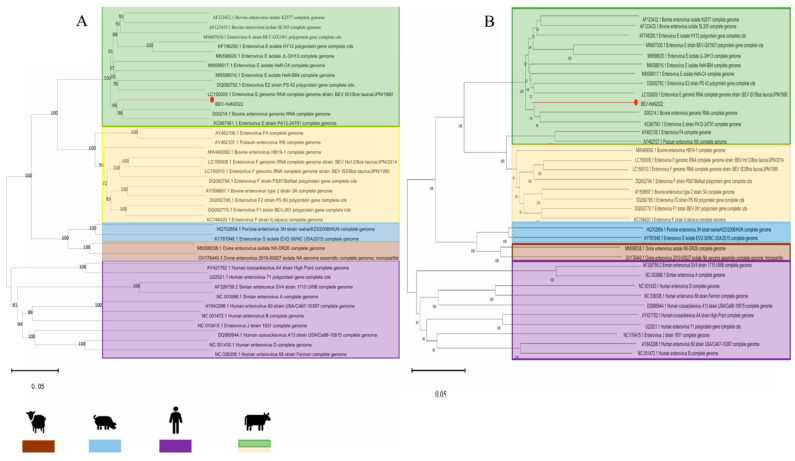
Phylogenetic analyses of the HeN-2022 strain. Phylogenetic trees were constructed using the neighbor-joining method with 1000 bootstrap replicates in the MEGA 11.0 software. The scale bars indicate the nucleotide substitutions at each site: (**A**) VP1 gene alignment of the HeN-2022 strain; (**B**) Complete genome sequence alignment of the HeN-2022 strain.

**Figure 4:**
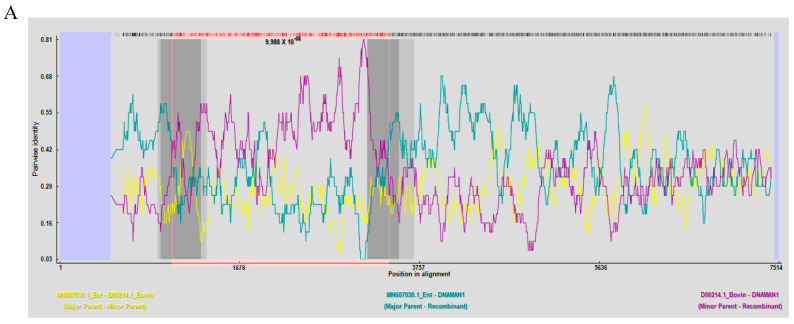

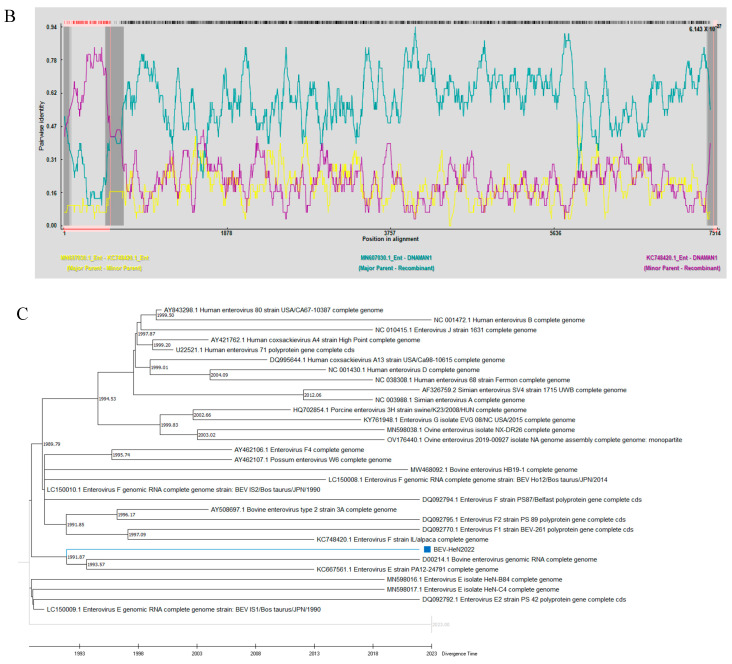
Viral recombination was analyzed and the potential recombinant breakpoints were identified by RDP4.0. BOOTSCAN on the basis of pairwise distance with 1000 bootstrap replicates. Recombination analysis of newly discovered complete genes of bovine enterovirus. (**A**): Predicted recombination events 1 with BEV−E4 (GX1901, China) and BEV−E1 (VG527, Ireland). (**B**): Predicted recombination events 2 with BEV−E4 (GX1901, China) and BEV-F1 (IL, USA). (**C**): The time tree was calculated in MEGA where divergence time was inferred by the RelTime with Dated Tips method. The HeN−2022 strain was designated as an outgroup taxon and all sequences used the year of sampling dates as the tip dates for calibration constraints.

The phylogenetic analysis of the VP1 gene sequence placed HeN-2022 within the EV-E1 clade, showing the closest affinity with the Irish strain VG527 (D00214) and USA BEV-E1 strain PA12 (Figure 3A). Additionally, phylogenetic analysis based on the complete genome demonstrates concordant genetic features with the VPl gene-specific profiling (Figure 3B). Recombination analysis using RDP 4.0 identified two statistically significant events (*p* < 0.01). The first event, VP1 structural gene recombination (nt 1224–3761), primarily originates from BEV-E1 (VG527, Ireland) and secondarily from BEV-E4 (GX1901, China), with a breakpoint in the receptor-binding canyon domain (Figure 4A). The second event, 5′ UTR Recombination (nt 24–643), involves BEV-E4 (GX1901, China) and BEV-F1 (IL, USA), possibly representing the first reported recombination event within the BEV-E1 genotype (Figure 4B). Divergence time estimation (RelTime-ML) traced the origin of HeN-2022 to 1991, dating back further than its closest relatives D00214 and PA12 (Figure 4C).

### 3.4. Cross-Species Infectivity in Sheep

**Figure 5:**
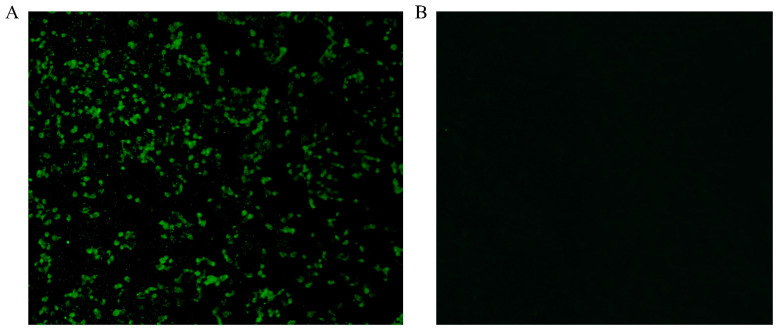
MDBK cells inoculated with tissues obtained from HeN-2022-infected sheep showed many BEV-positive cells detected by IFA at 36 hpi. (**A)** Positive sample;(**B**) Negative control.

The experimental infection of sheep revealed asymptomatic viral shedding, with fecal viral RNA peaking at 5 dpi. Seroconversion was confirmed by IFA, with neutralizing antibodies detectable by 7 dpi (Figure 5). No clinical symptoms were observed, indicating subclinical replication and adaptive immune activation.

## 4. Discussion

### 4.1. Global BEV Epidemiology and Emergence of HeN-2022

Bovine enteroviruses (BEVs) are significant pathogens associated with diarrheal and respiratory diseases in cattle, with reported prevalence rates varying globally—85.3% in Turkey, 69% in Spain, and 14.5% in Brazil between 2010 and 2022 [12,14,18,19]. In China, BEV prevalence has persisted at 10.7–27.6% over the past decade [20], with regional hotspots in Jilin (22.2%), Shandong (15.8%), and Guangxi (10.75%) provinces [21]. Despite declining national prevalence, localized outbreaks remain a threat to livestock health. Our study identified a BEV positivity rate of 19.2% in Henan Province, aligning with historical data and underscoring the necessity for sustained surveillance to mitigate future outbreaks [15,22].

The isolation of HeN-2022, a novel recombinant BEV strain, from cattle with severe diarrhea highlights the ongoing evolution of enteroviruses in China. Phylogenetic analysis positioned HeN-2022 within the EV-E1 clade, marking the first detection of this genotype in China. Its genetic proximity to Irish BEV−E1 strain VG527 and China BEV-E4 strain GX1901 suggests transcontinental transmission, likely facilitated by livestock trade or migratory patterns.

### 4.2. Recombination as a Potential Driver of BEV Evolution

Recombination is a pivotal mechanism for RNA virus diversification, enabling rapid host adaptation and zoonotic potential [14,15,23,24]. In the HeN-2022 strain, two recombination events were identified. The first event occurred in the VP1 structural gene (nt 1224–3761), involving BEV-E1 (VG527, Ireland) and BEV-E4 (GX1901, China). This recombination introduced amino acid substitutions in the receptor-binding “canyon” domain, potentially altering viral tropism. The second event was a 5′ UTR recombination (nt 24–643), derived from BEV-E4 (GX1901, China) and BEV-F1 (IL, USA), which may modulate viral replication efficiency.

The detection of HeN-2022 in Henan, which is geographically distant from Guangxi (the source of GX1901), implies the widespread dissemination of recombinant strains. This dissemination may occur via asymptomatic carriers. Such findings align with reports of BEV in bovine semen, implicating vertical transmission and potential reproductive complications [22].

### 4.3. Evolutionary Origins and Divergence Dynamics

Divergence time estimation traced HeN-2022 to 1991, predating its closest relatives (D00214 and PA12) [9,25]. This temporal gap suggests prolonged cryptic circulation, possibly in non-bovine reservoirs, before spillback into cattle populations. Moreover, the phylogenetic incongruence between the VP1 region (EV-E1 clade) and the 5′ UTR (EV-E4 origin) highlights the recombinant nature of HeN-2022. The VP1 phylogeny reflects its structural gene ancestry, while recombination events have introduced genomic changes from divergent lineages into the 5′ UTR. This genetic variation has facilitated adaptive evolution.

The early divergence of HeN-2022 relative to modern strains like PA12 suggests that the parental lineages involved in recombination existed as undetected variants for decades. These ancestral lineages may have persisted in non-bovine reservoirs or geographically isolated populations before spilling back into cattle. Notably, HeN-2022 clusters phylogenetically with PEVs, which may suggest the potential for cross-species transmission within the *Picornaviridae* family. However, the lack of identified intermediate hosts in this study precludes definitive conclusions about the specific transmission pathways. This highlights the need for more extensive surveillance efforts to better understand the evolutionary dynamics [26].

### 4.4. Challenges in BEV Pathogenicity Modeling

Animal models are critical for elucidating BEV pathogenesis, yet no universally accepted system exists [27]. While calves are traditional models, their high cost and ethical constraints have driven the exploration of alternatives. Additionally, BEVs are mainly transmitted via the fecal–oral route in natural settings. Viral particles shed in feces can contaminate feed, water, or the environment and are subsequently ingested by susceptible animals. Here, we employed both oral inoculation and intraperitoneal injection methods to investigate the infection dynamics of BEVs. Oral inoculation was used to closely mimic the natural infection route. However, oral inoculation can introduce variability due to the presence of gastrointestinal barriers, such as gastric acid and proteolytic enzymes, which may partially inactivate viral particles. To overcome this limitation, we also utilized intraperitoneal injection, which bypasses the gastrointestinal tract and facilitates consistent dissemination to target tissues.

In this study, sheep exhibited asymptomatic viral shedding (peak at 5 dpi) and seroconversion, mirroring subclinical infections observed in natural hosts. The age-dependent response—viral detection in adults but not lambs—aligns with immune maturation trends and suggests sheep as viable models for BEV persistence studies. However, the lack of pathological data (e.g., histopathology, cytokine profiling) limits in-depth exploration of the mechanisms, highlighting a need for integrated approaches in future work.

### 4.5. Implications for Surveillance and Control

The emergence of HeN-2022 exemplifies the risks posed by recombinant BEVs to livestock industries. Its dual recombination events, spanning structural and non-coding regions, emphasize the need for genomic surveillance to track emerging variants. Targeted vaccination strategies, focusing on conserved VP1 epitopes, could mitigate outbreaks, while biosecurity measures must address potential interspecies transmission routes

## 5. Conclusions

In this study, we identified and characterized a novel recombinant BEV, HeN-2022, isolated from cattle with diarrhea in Henan Province, China. Through comprehensive genomic and phylogenetic analyses, we revealed that HeN-2022 emerged from two distinct recombination events, with breakpoints located in the VP1 structural protein and 5′ UTR regions. Divergence time estimation traced the origin of HeN-2022 to 1991, potentially suggesting prolonged cryptic circulation prior to its detection. Experimental infection in sheep showed asymptomatic and robust seroconversion, highlighting the strain’s potential for cross-species adaptability without causing overt clinical disease. As the first reported BEV-E1 genotype in China, HeN-2022 highlights the significant role of recombination in driving BEV evolution and underscores potential risks associated with the transboundary dissemination of enterovirus. Our findings emphasize the necessity of integrated surveillance that combines genomics, phylogenetics, and animal modeling to preempt potential outbreaks in livestock populations.

## Data Availability

The original contributions presented in this study are included in the article/Supplementary Material. Further inquiries can be directed to the corresponding authors.

## Supplementary Materials

The following supporting information can be downloaded at: https://www.mdpi.com/article/10.3390/ani15101457/s1, Table S1: Strain information used for analysis. Table S2: Strain information used for analysis.
