# Supplementary material for: Emergence of a Recombinant Bovine Enterovirus in China: Insights from Phylogenetic and Temporal Analysis
Source: Animals (Basel). 2025 May 18;15(10):1457. doi: 10.3390/ani15101457 (PMC12108324; doi:10.3390/ani15101457)
Supplement: Supplementary file 1 [file animals-15-01457-s001.zip › animals-3571456-supplementary.pdf]

Supplemental materials

Table S1 Primers for PCR amplification of the BEV genome

Primer name	Product size（bp）	Sequence（5’-3’）
BEV-1F	1743	AGCCTGGGGGTTGTACCCACCCCTGG
BEV-1R		CCGAGCGTGTGCGCACAAACCAGTG
BEV-2F	1579	GAATGCTGCTAATCCCAACCTCCG
BEV-2R		GCACCAATGGATCAACCTAAGAACAAACAATTCG
BEV-3F	920	GGAAATTCACTTATATACCCGCACCAA
BEV-3R		GCCATAGACAAACAGGTGGCAGGAGCC
BEV-4F	1479	GCGCTGGAAGATAAGGGAAAACTCTTC
BEV-4R		AAGGCCAACCCACAGAGTCTTAATGTATGAG
BEV-5F	840	CGTTGGGCGCGTCAAAGATTA
BEV-5R		TTTAAGGCGCTCGAGAGGAAGTGGT

Table S2 Strain information used for analysis

Strain name	Accession NO.	Time	Location	Sub-Genotype
BEV-261	DQ092770	2005	German	EV-F1
IL-alpaca	KC748420	2013	USA	EV-F1
3A	AY508697	2004	USA	EV-F2
PS89	DQ092795	2005	German	EV-F2
Ho12	LC150008	2014	Japan	EV-F3
IS2	LC150010	2016	Japan	EV-F3
PS87	DQ092794	2005	German	EV-F3
HB19-1	MW468092	2021	China	EV-F3
W1	AY462106	2003	New Zealand	EV-F4
W6	AY462107	2003	New Zealand	EV-F4
VG527	D00214	1988	UK	EV-E1
A12-24791	KC667561	2013	USA	EV-E1
HeN-2022	OR058627	2022	China	EV-E1
IS1	LC150009	2016	Japan	EV-E2
PS42	DQ092792	2005	German	EV-E2
JL-DH12	MN598020	2019	China	EV-E3
HY12	KF748290	2013	China	EV-E3
SL305	AF123433	1956	Australia	EV-E4
GX1901	MN607030	2019	China	EV-E4
3H	HQ702854	2008	Hungary	EV-G
EVG08	KY76148	2015	USA	EV-G

NX-DR26	MN598038	2019	China	Ovine enterivirus
2019-00927	OV176449	2019	Germany	Ovine enterivirus
A4	AY421762	2003	USA	EV-A
EV71	U22521	1995	USA	EV-A
SV4	AF326759	2007	USA	EV-H
A-2	NC-003988	1999	USA	EV-H
B80	AY843298	2004	USA	EV-B
Uknown	NC-00147	2000	USA	EV-B
Uknown	NC-010415	2007	USA	EV-J
Uknown	DQ995644	2006	USA	EV-C
Uknown	NC-001430	2004	USA	EV-D
Uknown	NC-038308	2004	USA	EV-D
C4	MN598017	2019	China	EV-E3
B84	MN598016	2019	China	EV-E3
K2577	AF123432	1999	Australia	EV-E4
